# Mémoire Sur Les Causes Générales Des Syphilides, Et Sur Les Rapports, Qui Existent Entre Ces Affections Cutanées Et Les Symptomes Primitifs De La Maladie Vénérienne

**Published:** 1838-09-26

**Authors:** 


### BIBLIOGRAPHICAL NOTICE.

Mémoire *sur les Causes Générales des Syphilides, et sur les Rapports, qui existent entre ces Affections Cutanées et les Symptomes Primitifs de la Maladie Vénérienne.* Par C. F. Martins, D. M., &c., &c. Paris: 1838. 8vo. pp. 110.

A Memoir *on the General Causes of Syphilides, and the Relations which exist between these Cutaneous Affections and the Primary Symptoms of the Venereal Disease.* By C. F. Martins, M. D., &c. &c. Paris: 1838. 8vo. pp. 110.

The author of this monograph, during a residence of two years in the hospital of St. Louis, at Paris, collected forty-four observations of syphilitic cutaneous affections, which he afterwards analysed in conjunction with sixteen others, selected indifferently from cases reported by various writers on the venereal disease. With these materials he has attempted the solution of some interesting questions, involving the causes and nature of the secondary syphilitic eruptions, their relations with the primary affection, and the modifying influence which treatment, climate, and temperament, exercise upon their developement and progress.

As preliminary to his analysis, Dr. Martins adopts the belief in a venereal virus, not as a substantial existence, but as an hypothesis which serves to connect together the phenomena of syphilis. This seems to be an unmeaning distinction, but, without pausing to examine the speculations of the author, we pass at once to the more valuable portion of his work, the result of his observations. The syphilides, he informs us, were noticed by him, almost exclusively under the papular, tuberculous, pustular, and ulcerated forms. The exanthematous, squamous, and vesicular varieties, he rarely observed; and, as their real nature was involved in doubt, he preferred neglecting them altogether in his estimates.

The first important conclusion which Dr. Martins deduces, is, the analogous nature of the infection of blennorrhagia and chancres. According to him, they are both followed by the secondary cutaneous affections; blennorrhagia, however, less frequently than chancres. This identity of the two disorders is not, we think, satisfactorily established by the facts reported. The application of the speculum to the study of venereal diseases, has demonstrated the frequent cöexistence of masked chancres in the vagina and uterus, with the external symptoms of blennorrhagia. Chancres may, in the same manner, be concealed in the male urethra, and cases, presenting the appearance of simple blennorrhagia, may actually be complicated with one or more internal chancres. As Dr. Martins reports no case of blennorrhagia in a female, in which the absence of chancres was ascertained by the speculum, and in which secondary symptoms followed, we cannot admit that he has proved blennorrhagia to be a cause of constitutional syphilitic infection.

On the subject of the relative frequency of particular syphilitic eruptions, Dr. Martins' observations establish that the papular syphilides are the most common, and the pustular the least frequent of the varieties. In more than one-half of the cases, the syphilides show themselves in the first instance on the face and hairy portions of the body.

The influence which the treatment of the primary symptoms exerts upon the developement and nature of the secondary cutaneous affections, is inappreciable. Mercury has no prophylactic virtue in preventing constitutional symptoms. Individuals of feeble, lymphatic, or scrofulous constitutions, are more frequently affected with syphilides than others, in the proportion of nineteen to thirteen. The complication of chancres with buboes, hastens the period of the appearance of secondary symptoms, and the probability of their occurrence.

The duration of a chancre has no effect in hastening or retarding the cutaneous eruption, according to Dr. Martins. It must, we, however, think, exercise a material influence in determining the probability of the developement of constitutional symptoms. Proportionate to the rapidity with which the chancre is cured, must be the diminution of the risk of the absorption of the virus into the system.

The pustular syphilides usually first succeed the symptoms of infection; the average lapse of time, after the primary affection, at which they showed themselves, in Dr. Martins' cases, was seven months. Next in order are the papular syphilides, after an average lapse of twenty-one months, the tuberculous, after five years, the ulcerated, after eight years, and the tuberculo-ulcerated, after eight years and a half. The gravity of the secondary affections does not appear to be mitigated by the length of the interval which separates them from the primary. After the age of thirty-four, the chances of being affected with secondary syphilis are diminished at least one-third.

The varieties of temperature, Dr. Martins tells us, exercise a sensible influence upon the developement of syphilides. Heat is more favourable to to their appearance than cold. The effect of a low temperature, as 26° Fahrenheit, is nearly as powerful, in this respect, as that of one of moderate heat, say of 60°. A temperature of about 43° seems most likely to prevent the occurrence of syphilides. The influence of temperature is rapidly felt, artificial and atmospheric extremes producing similar effects. Finally, all causes which tend to enfeeble the system, as illness, medical or surgical, non-syphilitic cutaneous eruptions, fatigue, moral emotions, determine the developement of the syphilides.

We have presented the most important conclusions which Dr. Martins has derived from the analysis of his observations. We cannot admit that his facts bear him out in all his deductions, but many of them are unquestionably both novel and interesting. It is to be regretted that he was unable to accumulate a greater mass of materials for analysis, and to include some cases of females in the subjects of his investigations. This omission, as we have pointed out, has rendered his observations defective in, at least, one important particular. But, as the Doctor well remarks, much time and much labour are required to collect even forty cases of chronic disease. The faithful and careful observation of this amount of facts, is no trifling contribution to medical science.

